# Primary Plasmacytoma of the Kidney

**DOI:** 10.1155/2013/239580

**Published:** 2013-07-01

**Authors:** R. A. J. Spence, A. Thwaini, D. M. O'Rourke

**Affiliations:** ^1^Department of Urology, Belfast City Hospital, 51 Lisburn Road, Belfast BT9 7AB, UK; ^2^Department of Pathology, Belfast City Hospital, 51 Lisburn Road, Belfast BT9 7AB, UK

## Abstract

Primary renal plasmacytomas are an extremely rare clinical condition. Their management is particularly challenging due to the paucity of evidence, with only just over a dozen previously reported cases. We report a case of a primary extramedullary plasmacytoma of the kidney and performed a review of the literature. The case is presented as a learning point that it is imperative to keep plasmacytic tumours in mind and to include them in the differential diagnosis of anaplastic tumours, even in unusual locations, such as the kidney.

## 1. Introduction

Plasmacytomas are malignant plasma cell tumours which are characterised by the proliferation of a single clone of plasma cells, producing monoclonal immunoglobulins. They may arise in soft tissue (extramedullary), or within the skeleton [1]. Skeletal plasmacytomas are the most common primary, with extramedullary plasmacytomas typically arising from fat, muscle, or mucosal surfaces; common sites include respiratory and digestives tracts, head and neck regions, with more than 80% situated above the diaphragm [2, 3]. Extramedullary plasmacytomas typically affect patients during middle age (median 55–60 years) and are more common in males (Male : Female 3 : 1) [4]. Confirmed risk factors for plasmacytomas remain unknown; however, prior radiation exposure has been suggested [5]. 

## 2. Case Report

**Figure 1:**
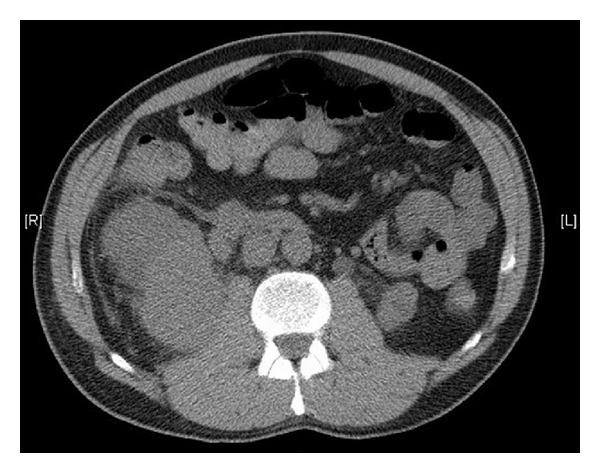
Axial CT image demonstrating primary plasmacytoma of the kidney.

**Figure 2:**
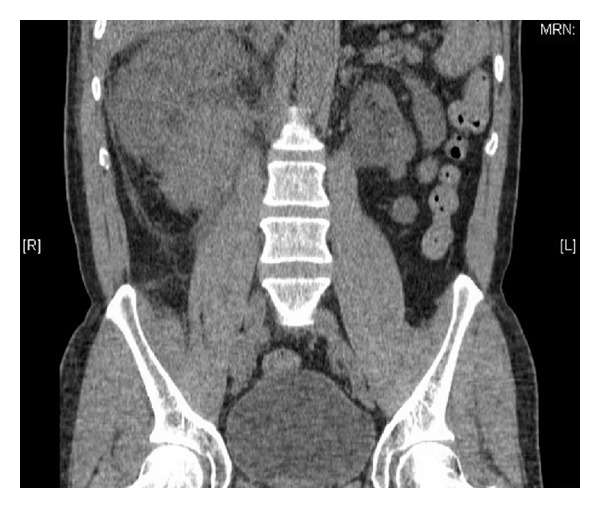
Coronal CT image demonstrating primary plasmacytoma of the kidney.

A 49-year-old male presented with a two-day history of sudden, severe, right sided loin pain which was associated with vomiting. There was no history of any prior lower urinary tract symptoms. Past medical history included squamous cell carcinoma of the tongue for which the patient underwent partial excision and adjuvant radiotherapy and had no evidence of recurrence. Haematological investigations revealed deranged renal function with significantly raised serum creatinine. C-reactive protein was also elevated.

(Computed tomography) CT and (magnetic resonance) MR imaging demonstrated a solid lesion involving the lower two thirds of the right kidney (Figures 1 and 2). The left kidney was atrophic. The patient was initially managed with right JJ stenting, which resulted in normalisation of his creatinine. However, renal function began further deterioration, thus underwent right percutaneous nephrostomy tube insertion. Renal function normalised following this procedure.

**Figure 3:**
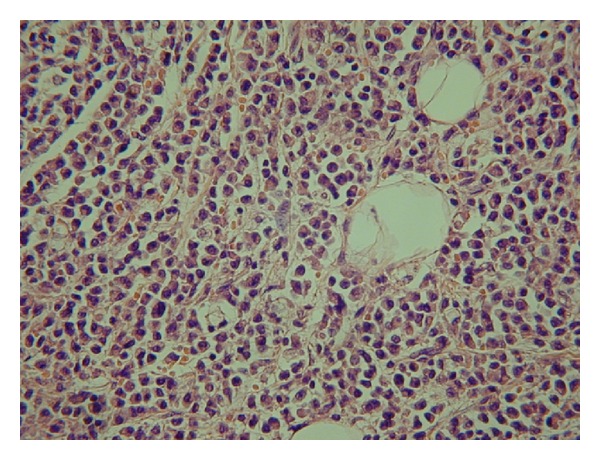
Histopathology of primary plasmacytoma of the kidney.

As imaging demonstrated that this lesion was atypical, a renal biopsy was performed. Results were highly suggestive of a primary extramedullary plasmacytoma of the kidney (Figure 3). Plasma electrophoresis for paraproteins was normal. In addition, both urinary Bence-Jones proteins and bone marrow biopsy returned normal.

Following multidisciplinary team discussion, it was decided that initial management was surgical, with an attempt to perform a partial nephrectomy. The procedure was challenging due to gross involvement of the surrounding tissues and tumour adhesion to the psoas muscle, which resulted in proceeding to right radical nephrectomy. Haemodialysis was required following surgery.

There was a smooth postoperative recovery. Histology demonstrated anaplastic extramedullary plasmacytoma of the kidney. 

## 3. Discussion

A primary renal extramedullary plasmacytoma is a rare clinical condition with just over a dozen cases reported in the literature. The largest series was reported from the Mayo Clinic [6]. In their series, they demonstrated that renal plasmacytomas could mimic renal cell carcinomas, or even transitional cell carcinomas of the kidney. In addition, they confirmed that anaplasia of tumour cells does not necessarily portend a poor prognosis [6].

It is of importance to note that plasmacytoma may be composed of pleomorphic cells with little resemblance to normal plasma cells. These tumours require immunohistochemistry with addition to electron microscopy for accurate diagnosis. Therefore, it is imperative to keep plasmacytic tumours in mind and include them in the differential diagnosis of anaplastic tumours, even in unusual locations, such as the kidney [7].

 In cases of plasmacytoma, lymph node involvement can be a manifestation of the disease either at presentation, or relapse. This normally occurs in approximately 40% of cases [8]. However, in our case there was no evidence of lymph node involvement. It is also important to recognise that in cases of extramedullary plasmacytoma, protein electrophoresis shows a monoclonal component in only 25% of cases; therefore, it is essential not to exclude plasmacytoma merely based on negative laboratory tests [3]. 

## 4. Conclusion

This case represents a rare example of extramedullary plasmacytoma of renal origin. It is important to consider all possible differential conditions when evaluating renal masses. Due to rare nature of the disease, there is paucity of the available evidence in the literature.
